# Dynamic Diversity of Glial Response Among Species in Spinal Cord Injury

**DOI:** 10.3389/fnagi.2021.769548

**Published:** 2021-11-26

**Authors:** Jean-Christophe Perez, Yannick N. Gerber, Florence E. Perrin

**Affiliations:** ^1^MMDN, Université de Montpellier, EPHE, INSERM, Montpellier, France; ^2^Institut Universitaire de France (IUF), Paris, France

**Keywords:** spinal cord injury (SCI), glial cells, immune cells, glial scar, glial bridge, rodents, primates, regenerative species

## Abstract

The glial scar that forms after traumatic spinal cord injury (SCI) is mostly composed of microglia, NG2 glia, and astrocytes and plays dual roles in pathophysiological processes induced by the injury. On one hand, the glial scar acts as a chemical and physical obstacle to spontaneous axonal regeneration, thus preventing functional recovery, and, on the other hand, it partly limits lesion extension. The complex activation pattern of glial cells is associated with cellular and molecular crosstalk and interactions with immune cells. Interestingly, response to SCI is diverse among species: from amphibians and fishes that display rather limited (if any) glial scarring to mammals that exhibit a well-identifiable scar. Additionally, kinetics of glial activation varies among species. In rodents, microglia become activated before astrocytes, and both glial cell populations undergo activation processes reflected amongst others by proliferation and migration toward the injury site. In primates, glial cell activation is delayed as compared to rodents. Here, we compare the spatial and temporal diversity of the glial response, following SCI amongst species. A better understanding of mechanisms underlying glial activation and scar formation is a prerequisite to develop timely glial cell-specific therapeutic strategies that aim to increase functional recovery.

## Introduction

Traumatic injuries, including spinal cord injury in the adult mammalian central nervous system, induce a glial response that eventually forms a glial scar that is largely occupied by microglia, NG2 glia and astrocytes. The first glial cells to be activated, after injury, are microglia/macrophages that either proliferate and migrate toward the lesion site or, in the case of monocyte-derived macrophages, infiltrate from the periphery. The activated microglia/macrophages concomitantly express a full repertoire of molecules that modulate glial responses (including microglia/macrophages) but also immune-cell responses (for review, see David and Kroner, 2011; David et al., 2015, 2018). The response of astrocytes eventually leads to the formation of a dense astroglial border surrounding the lesion core, or fibrotic scar (for review, see Yang et al., 2020). In the past decade, the concept that the glial scar has both harmful and beneficial effects has emerged. Indeed, the scar acts as a chemical and physical obstacle to spontaneous axonal regeneration and thus prevents functional recovery. However, the glial scar also limits lesion extension. A better understanding of the complexity of individual cellular (glial and immune cells) and molecular mechanisms induced by SCI as well as their crosstalk remains a major challenge. The cellular dynamics induced by injury are closely reflected by tissue repair and functional recovery. Remarkably, amphibians and fishes (for review, see Ghosh and Hui, 2018), but also embryonic/neonatal mammals, exhibit the capacity to both repair injured spinal cord tissues and to achieve functional recovery. Interestingly, these animals display rather limited (if any) glial scarring.

Here, we review the temporal diversity of the glial response, following SCI in rodents, primates, and species that display high regenerative capabilities. Due to the abundant literature on glial scarring, especially in rodents, we selected articles mainly focusing on descriptive characterisations of cellular and/or temporal events induced by SCI in order to highlight the consequences of glial-scar formation kinetics on functional recovery after injury. A better understanding of the mechanisms underlying the time line of glial activation and scar formation is a prerequisite to develop glial-cell-specific therapeutic strategies.

## Mice: A Major Glial Scar Is Observed After Spinal Cord Injury

**FIGURE 1:**
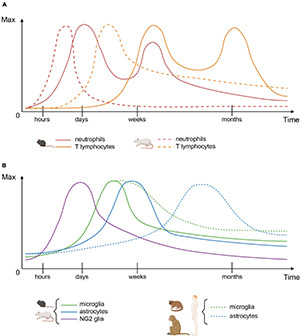
Cellular dynamics after spinal cord injury. **(A)** Immune cell infiltration patterns in mice (plain lines) and rats (dashed lines). **(B)** Glial cell numbers in rodents (plain lines) and primates (dashed lines). For each cell type, both graphs represent the number of cells over time, relative to their maximum value.

**FIGURE 2:**
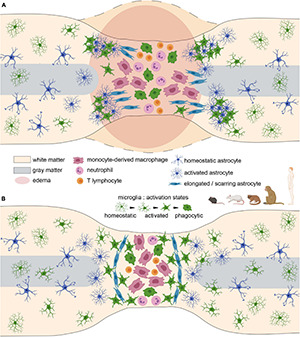
Glial scar formation after spinal cord injury in rodents and primates. **(A)** Acute stage. Cellular infiltration, reactivity, proliferation, and edema at the lesion site. **(B)** Glial scar stabilisation at the subacute/chronic stage. Note the substantial role of scarring astrocytes in separating the lesion core from spared tissues.

Owing to the extensive availability of genetically modified animals, mice are the most widely used model to study the cellular and molecular responses of glia following SCI (Figures 1, 2 and Table 1).

**TABLE 1:** Studies demonstrating roles of the glial and immune cells after SCI in mice.

**Injury, interval SCI-death, methods**	**Astrocyte**	**Microglia/macrophage**	**Other glial cells**	**Immune cells**	**References**
**Mice**					
Contusion T9. 3, 7, 21, 28&42dys. IHC		CD11b MHCII		CD3,CD4, CD8	Sroga et al., 2003
HS T8. 8, 30, 90, 180&365dys: IHC	GFAP PSA NCAM	Isolectin B4	NG2		Camand et al., 2004
Contusion T9. 3, 7, 14&42dys, IHC		Mac1, MHCII		LY6G,CD3, CD4, CD8	Kigerl et al., 2006
Contusion T10-11. 15&45mns, 3&24hrs, 2&14dys	GFAP	Iba1, CD11b	CA2	CD45	Pineau and Lacroix, 2007
Contusion T9-10. 3, 7&28dys. Microarrays, IHC		CD86, CD206, CD16, CD32, Arginase1			Kigerl et al., 2009
Contusion T10-11. 3&12hrs, 4&28dys IHC, FACS	GFAP	Iba1. FACS: CD11b, CD45, CD16, CD32	CA2	7/4, LY6B FACS: F480, LY6C, LY6G,	Pineau et al., 2010
Contusion T9. 3, 7&49dys BrdU, IHC	GFAP BLBP	CD11b			White et al., 2010
Compression T5. 1, 3, 7, 14&42dys.				LY6G F480 Tg: LysM	Mawhinney et al., 2012
Dorsal HS T9. 1, 4&54dys. Microarrays, BrdU	GFAP	CD11b	NG2		Thuret et al., 2012
Dorsal section C4. 7dys&14wks TgFoxJ1, IHC	GFAP				Sabelstrom et al., 2013
Crush L1-2. 5, 14&28dys TgSTAT3KO, BrdU, IHC	Tg: GFAP GFAP Aquaporin4 BLBP, RC2	CD45	SOX2		Wanner et al., 2013
Contusion T11. 24hrs, 3, 7, 14&42dys. IHC		Iba1, CD11b			Greenhalgh and David, 2014
Laser injury. 5, 30&120mins, FACS		Tg: CX3CR1 CD11^+^/Ly6C^+^ CD45			Stirling et al., 2014
HS T12. 30mins, 2, 8, 24, 48&72hrs. IHC		F480			Tang et al., 2015
Contusion T8. 3, 5, 7, 14, 28&56dys. IHC	GFAP	Tg: CX3CR1 CD11b		Tg: LysM	Zhu et al., 2015a
Crush T10. 2, 8&10wks RNAseq. Transgenic STAT3 KO, BrdU	Tg: GFAP GFAP		NG2		Anderson et al., 2016
Lateral crush T8. 3, 5, 7&14dys.	Tg: GFAP GFAP	CD11b	CC1		Fan et al., 2016
HS and FT T9. 1&2wks. FACS, RNA-seq, IHC	Tg: Aldh111 GFAP, FGFR4				Noristani et al., 2016
HS and FT T9. 72hrs, 1&2wks. FACS, RNA-seq, IHC	GFAP, Vim	Tg: CX3CR1 Iba1			Noristani et al., 2017
Crush and lateral stab T10. 2&8wks. Tg FoxJ1, BrdU, IHC	GFAP, Aldh111				Ren et al., 2017
Contusion T8. 3&7dys. RNAseq, IHC	GFAP			Tg: LysM Tg:CD45, Tg: CD36	Zhu et al., 2017
Contusion T11. 1, 3, 4, 7&28dys. IHC.		CD11b, CD86, Iba1,P2RY12, TMEM119		Tg: LysM Tg: CCR2	Greenhalgh et al., 2018
Lateral contusion C5. 1, 3, 7, 11, 14&21dys, IHC	GFAP		Tg: NG2 ablation Olig2		Hesp et al., 2018
Contusion T9-10 1, 4, 7, 14&35dys, IHC	GFAP SOX9	R26-TdT Tg: LysM Tg: CX3CR1^cre^ CD68,P2RY12,			Bellver-Landete et al., 2019
Crush T8. 2, 4&6wks Lentiviral-induced ablation, BrdU, IHC	Lv-GFAP to ablate astrocytes	Iba1			Gu et al., 2019
Crush T10 3&7dys, 10wks,IHC, RNA-seq.	GFAP	CD68, P2Y12 RNA: CD11bTg: CX3CR1^cre^ Tg: CSF1R^fl/fl^			Li et al., 2020

*FACS, flow cytometry; hrs, hours; min, minutes; dys, days; wks, weeks; mths, months; yrs, years; IHC, immunohistochemistry; C, cervical; T; thoracic; L, lumbar; HS, hemisection; FT, full transection; Tg, transgenic.*

In mice, immune-cell responses to SCI play a key role in the dynamics of the lesion. The recruitment of neutrophils, following contusion injury, displayed similar kinetics in four mouse strains. An early infiltration, starting as early as 6 h after injury, led to a peak of neutrophil number between 3 and 14 days post injury (dpi). This was followed by a decrease over the next 4 weeks. Neutrophil numbers, however, remained stable over the next 6 weeks of the study (Kigerl et al., 2006). Compression injury led to similar neutrophil kinetics with two waves of activation that peaked at 3 and 14 dpi (Mawhinney et al., 2012). Consistently, 3–12 h after contusion injury, expression of chemokines, such as KC (CXCL1) and MIP-2 (CXCL2) by astrocytes, was followed by the recruitment of neutrophils [and, to a lesser extent, monocytes] through MyD88/IL-1R1 signaling within damaged areas (Pineau et al., 2010). Analysis of the dynamics of cytokine expression after contusion injury has led to the suggestion that the early production (5–15 min) of IL-1β by astrocytes and microglia after injury orchestrates the recruitment of leukocytes (Pineau and Lacroix, 2007). Subsequently, the release of IL-1β and TNF-α (14–28 dpi) induces the recruitment of T lymphocytes (Pineau and Lacroix, 2007). This is in agreement with the biphasic T-cell influx reported after contusion injury, starting at 14 dpi, and then decreasing between 2 and 4 weeks and again increasing over the following 2 weeks to reach similar number as at 14 dpi (Kigerl et al., 2006).

Microglia and macrophages are the two predominant immune players in SCI. Resident microglia are within the spinal cord before injury, whereas the monocyte-derived macrophages (MDM) infiltrate the spinal cord from the periphery after the lesion. Crosstalk between both cell types modulates their respective responses to injury and, therefore, contributes to their functions. The dynamic orientation of microglial processes toward the lesion, within the white matter, has been observed by time-lapse two-photon imaging as early as 5 min after laser injury. This led, soon afterward, to the initiation of myelin debris phagocytosis (Stirling et al., 2014). Similarly, in several mouse strains, contusion and compression injuries induced an early macrophage activation at 6 h postlesion that further formed phagocytic clusters in the grey matter by 3 dpi (Kigerl et al., 2006; Mawhinney et al., 2012). At 1 day post contusion, microglia rapidly accumulated around the epicenter but decreased in number (cell death, partly by apoptosis) and retracted their processes at the lesion site (Bellver-Landete et al., 2019). From 4 dpi, microglia displayed a round shape and started to express phagocytic markers (Bellver-Landete et al., 2019). From 4 (Bellver-Landete et al., 2019) or 7 (Kigerl et al., 2006; Mawhinney et al., 2012) to 14 dpi, activated microglia and MDM peaked and then decreased but remained elevated for up to 6 weeks (Kigerl et al., 2006).

Microglia primarily and transiently proliferated after two severities of spinal cord section, as reflected by an upregulation of genes associated with proliferation at 3 days but not at 7 and 14 days after injury (Noristani et al., 2017). Consistently, after spinal cord contusion, Ki67 expression was observed in 50% of microglia at the lesion epicenter at 4 dpi; the peak of microglia proliferation occurred at 7 dpi and only few (2–6%) Ki67^+^ microglia persisted at 14 and 35 days (Bellver-Landete et al., 2019). Additionally, microglia proliferated in greater numbers than infiltrating macrophages, and they initiated phagocytosis of damaged axons at 1 dpi (Bellver-Landete et al., 2019). Conversely, infiltrating macrophages started to phagocytose debris at 3–5 dpi and then progressively became the main phagocytic cells in the lesion and persisted chronically (up to 42 dpi) (Greenhalgh and David, 2014). In addition, the infiltrating macrophages repressed microglia-mediated inflammation and phagocytosis (Greenhalgh et al., 2018). Subsequent to proliferation, microglia were rapidly recruited around the lesion site and accumulated in the core of the lesion 3 days after hemisection (Tang et al., 2015). Similarly, 3 days after spinal cord contusion, CD11b^+^ cells first occupied the periphery of the injury site before being preferentially located in the lesion site 5–56 dpi (Zhu et al., 2015a). Microglia, surrounding infiltrating cells, were located at the interface between infiltrating leukocytes and astrocytes, forming an immune interface through their interaction with both GFAP^+^ astrocytes and blood-derived cells (Bellver-Landete et al., 2019). This “microglial scar” mostly visible from 14 to 35 dpi limited the spread of infiltrating cells outside of the lesion core and expressed IGF-1 that further promoted astrocytic proliferation and astrocytic scar formation (Bellver-Landete et al., 2019).

Analysis of activated microglia/macrophages revealed that, from 3 to 12 months post-injury, few cells were located in the core of the lesion as compared to the glial scar (Camand et al., 2004).

Finally, at a transcriptomic level, microarray experiments on spinal cord segments, centered on the contusion site, have revealed an induction of pro- and anti-inflammatory genes from 1 to 28 dpi. However, the upregulation of anti-inflammatory genes was more transient (up to 7dpi) than the pro-inflammatory genes (up to 1 month) (Kigerl et al., 2009). Three days after contusion injury, macrophage-specific transcriptomic analysis revealed an expression profile characteristic of cell migration that further evolved at 7 dpi to a typical profile of foam cells (Zhu et al., 2017). Lastly, using RNAseq of microglia/macrophages (CX3CR1^+^ cells), following partial and complete spinal cord section, we have shown that microglial activation is dependent on the time post-injury but not on the lesion severity (Noristani et al., 2017). Indeed, the transcriptomic profile at 3 dpi reflected cell proliferation and was associated with neuroprotective genes, whereas, in the 7 and 14 dpi, the profile switched to neuroinflammation-associated gene expression. Interestingly, from 3 to 42 dpi, over 6% of microglia expressed astrocytic markers [glial fibrillary acidic protein (GFAP) and vimentin] that may reflect an SCI-induced glial differentiation (Noristani et al., 2017).

Astrocytes play a central role in the formation of the glial scar, following CNS injury. Five days after moderate spinal cord contusion, astrocytes, identified by their expression of GFAP, were seen in the vicinity of the lesion. From 7 dpi, astrocytes formed an astroglial scar surrounding the injury site that stabilised at 14 dpi (Zhu et al., 2015a). In the longer term (56 days after lesion), GFAP^+^ cells were no longer observed in the lesion core (Zhu et al., 2015a). Likewise, 5–14 days following crush injury, astrocyte proliferation, together with the overlapping of astrocytic processes, started to form a dense scar. By 2 weeks postinjury, scar borders surrounded the lesion and restricted fibrotic and inflammatory cells to the core of the injury site, mainly included newly proliferative astrocytes. This “corral” organisation is STAT3 dependent (Wanner et al., 2013). In mice with spinal crush injury, selective ablation of scar-forming, reactive, and proliferating astrocytes hindered glial scar formation and led to an extensive influx of IBA1-positive microglia/macrophages. These findings highlight the constant cross-talk between glial cells and strongly suggest that reactive astrocytes modulate microglia/macrophage number and infiltration (Gu et al., 2019). This is consistent with the increased number of proliferating microglia observed at 109 days after hemisection in adult MRL/MpJ mice that possess exceptional regeneration capabilities, which do not form a scar after injury and display a reduced astrocytic response (Thuret et al., 2012).

Eight days after dorsal hemisection of the spinal cord, an overall orientation of astrocytic processes within the rostro-caudal axis was observed immediately adjacent to the lesion. The core of the lesion, with only few astrocytes, remained rather wide from 8 days to 1 month after lesion and diminished by 50% from 3 to 12 months, following injury (Camand et al., 2004). In the vicinity of the lesion, hypertrophic astrocytes, displaying the classical “stellate shape,” were present up to 6 months after injury. Thereafter, GFAP expression returned to a baseline value 6–12 months after injury (Camand et al., 2004). At 3–7 dpi after lateral crush injury, cavity-surrounding, reactive astrocytes have been shown to die by necroptosis. Moreover, induction of necroptotic, astrocytic markers partly resulted from the polarisation of M1 microglia/macrophages (Fan et al., 2016). Strikingly, 8–30 dpi, intense chondroitin sulfate proteoglycans (CSPG) expression was observed in astrocytes. This later almost disappeared. In parallel, PSA-NCAM, which is expressed by astrocytic end feet in the intact spinal cord, was increased in a subpopulation of reactive astrocytes from 8 to 30 dpi. This expression remained elevated at later time points (Camand et al., 2004). After severe crush injury, Cspg5 (neuroglycan C) and Cspg4 (NG2) were upregulated in scar-forming astrocytes. Furthermore, both NG2 and CSPG5 proteins were observed in the glial scar (Anderson et al., 2016), suggesting that astrocytes also participated in extracellular matrix dynamics.

The origin of scar-forming astrocytes remains to be elucidated. Newly formed astrocytes accumulated at the edge of the lesion by 7 days after moderate contusion injury and then remained at a constant level up to 49 days. Similarly, amongst the proliferative cells, an increased proportion of astrocytes was observed in the spared white matter (White et al., 2010). In parallel, radial glial cells (BLBP^+^) presented an early and sustained increase in incidence at the edge of the lesion and in the preserved white matter conversely to their transient presence in the spared grey matter and central canal (White et al., 2010). There is an ongoing debate as to the origin of the newly proliferative scar-forming astrocytes. Indeed, scar-forming astrocytes were either reported to mainly (Sabelstrom et al., 2013) or minimally (Ren et al., 2017) originate from ependyma-derived progeny. This discrepancy on the ependymal contribution to newly scar-forming astrocyte may depend on whether or not the ependyma was directly damaged by the primary injury (Ren et al., 2017). Finally, we investigated astrocytic plasticity overtime using RNAseq analysis of a pure population of astrocytes, following hemi- or complete spinal cord section and demonstrated a time and severity-dependent deregulation of gene expression. However, in both injury severities, over 10% of mature (as opposed to newly formed) astrocytes underwent an injury-induced trans-differentiation toward neuronal progenitors (Noristani et al., 2016; Noristani and Perrin, 2016).

Finally, two NG2-expressing cell populations (glial cells and pericytes) also participate in scar formation. From 1 to 11 days after contusion injury, dividing oligodendrocyte progenitors, the NG2^+^ glial cells, strongly outnumber dividing NG2^+^ pericytes and were restricted at the lesion border and in the spared tissue (Hesp et al., 2018). From 8 days to 6 months, an increased expression of NG2 was also reported in the glial scar, following hemisection; it returned to control value 1 year after injury (Camand et al., 2004). Interestingly, ablation of NG2^+^ cells induced a less-dense astrocytic border associated with macrophages infiltration (Hesp et al., 2018).

Overall, in mice, recruitment and infiltration of immune cells precede microglial and astrocytic responses (Figure 1). However, a complex molecular crosstalk between all cell populations orchestrates the formation of a well-defined and dense glial scar (Figure 2 and Table 1).

## Rats: A Major Glial Scar Is Also Observed After Spinal Cord Injury But Immune Infiltration Appears Earlier Than in Mice

Rats display an overall pathophysiological response to SCI that mimics some features of the human response, such as the formation of cavities. This is not observed in mice. Rats are thus the most widely used model in SCI even if they are not predominant amongst rodents in studies focusing on glia (Figure 2 and Table 2).

**TABLE 2:** Studies demonstrating roles of the glial and immune cells after SCI in rats.

**Injury, interval SCI-death, methods**	**Astrocyte**	**Microglia/macrophage**	**Other glial cells**	**Immune cells**	**References**
**Rats**					
Partial section, 1, 3, 6, 12, 24hrs and 2, 4, 8, 14&12wks. IHC, HC	GFAP	CD11b, ED1		Cresyl violet	Dusart and Schwab, 1994
Contusion T8, 12, 72hrs, 7, 28dys IHC	GFAP	CD11b, ED1, MHCII		CD5	Popovich et al., 1997
Stab dorsal 1&4mths	GFAP				Liesi and Kauppila, 2002
Contusion T9, 3, 7, 21, 28&42dys IHC		CD11b, MHCII		CD4, CD8, CD11c	Sroga et al., 2003
Contusion T8 1, 3&7dys, 6wks BrdU, IHC	GFAP	CD11b	NG2 CC1		Zai and Wrathall, 2005
Moderate contusion T8 3, 7, 28 &70dys			NG2 P75 P0		McTigue et al., 2006
Dorsal funiculotomy T8. 1hr, 10&30dys IHC	GFAP	CD11b ED1 CD68	Olig2		Wang et al., 2009
Contusion T8 (3 severities) FACS: 0–10dys, 14, 90&180dys, IHC; 1, 7, 14&90dys		FACS:ED1, CD11b IHC: ED1		FACS&IHCCD3, PME	Beck et al., 2010
Dorsal HS 3, 7, 14&28dys, IHC		ED1, CD8, CD86, CD206		MPO, CD43	Pruss et al., 2011
ContusionT8 56dys, IHC	GFAP				Zhu et al., 2015b
FT T8 2, 8wks IHC	Morphology				Li et al., 2018
FT T9 48hrs IHC			Nr3c1, ependymal glia is a Glcc target		Nelson et al., 2019

*FACS, flow cytometry; hrs, hours; min, minutes; dys, days; wks, weeks; mths, months; yrs, years; IHC, immunohistochemistry; HC, histochemistry, H&E, hematoxylin eosin; C, cervical; T, thoracic; L, lumbar; HS, hemisection; FT, full transection.*

In rats, following spinal cord injury, the cellular response in the lesion is initiated by immune cells. The majority of studies have been carried out using immunohistochemistry, and only a few have resorted to flow cytometry. As early as 1–3 h, following partial spinal cord section, a few neutrophils adhere to the inner surface of blood vessels. Then, from 6 to 24 h, a large number of neutrophils are found at the site of the primary lesion. Thereafter, they disappear (Dusart and Schwab, 1994). Similarly, 1 day following contusion injury, the initial phase of inflammation consisted of an early neutrophil number peak that declines afterward. However, neutrophils persist for many months, and a positive correlation between contusion severity and the number of neutrophils has been reported (Beck et al., 2010). Finally, neutrophil and lymphocyte peaks were observed 3 days after dorsal hemisection of the spinal cord; neutrophils completely disappeared 7 days after lesion, whereas T cells displayed a strong decrease but remained present (Pruss et al., 2011). In agreement with this, following contusion injury, early T cell infiltration peaked between 3 and 7 dpi (Popovich et al., 1997; Sroga et al., 2003) and declined by 50% over the next 3 weeks (Sroga et al., 2003). Lymphocyte infiltration was paralleled by microglial activation (Popovich et al., 1997) and dendritic-cell influx (Sroga et al., 2003). Using flow cytometry, after contusion injury, Beck et al. show similar T cells dynamics, but with a slightly delayed infiltration (from 7 to 9 dpi peaking at Day 9), followed by a decrease at 10 dpi and persistence throughout the 6 months study follow-up (Beck et al., 2010).

Glial cell dynamics, including microglia/macrophages, oligodendrocytes, astrocytes, and NG2-expressing cells, have been widely analysed in rat models of SCI. The partial section of the spinal cord first induced microglia/macrophage proliferation at the lesion site that predominated at 48 h, leading to a highest density between 4 and 8 dpi. Then, 2 weeks after injury, microglia progressively disappeared from the lesion site concomitantly with the formation of a cavity that was further surrounded by a scar composed of microglia and astrocytes (Dusart and Schwab, 1994). Similarly, microglial activation peaked within the contusion epicenter between 3 and 7 days (Popovich et al., 1997; Sroga et al., 2003) and plateaued between 7 and 28 dpi distal to the lesion (Popovich et al., 1997). Alongside, monocyte influx and macrophage activation started at 7 dpi (Popovich et al., 1997).

The number of contusion-induced microglia/macrophages increased with the injury severity and displayed a biphasic response, with a first peak at 7 dpi, followed by a very low cell number at 14 dpi, increasing to a second peak at 60 days; microglia/macrophage number then remained elevated throughout 180 dpi (Beck et al., 2010). In agreement with this, a peak of microglia/macrophages displaying thick and branched processes was observed 1 week after dorsal hemisection, followed by a slow decline in number; however, microglia/macrophages also remained elevated 70 days after the lesion (Pruss et al., 2011). Following contusion injury, microglia/macrophages located in the spared white matter proliferated from 1 to 7 days, reaching a maximum on Day 3. By 6 weeks postlesion, few remaining proliferative microglia/macrophages were present (Zai and Wrathall, 2005). Finally, after dorsal funiculotomy, in ascending and descending pathways undergoing Wallerian degeneration at both subacute (10 dpi) and chronic (30 dpi) stages, the numbers of microglia (OX42+) and macrophages (ED1^+^) were higher than in sham animals. However, a decrease in cell number between subacute and chronic stages was seen only in the ascending tract (Wang et al., 2009). In the same animals, the number of astrocytes was also increased, *cf.* sham animals, at both stages but, conversely to microglia, remained stable between stages (Wang et al., 2009).

One week after dorsal hemisection, few astrocytes were located in the lesion site; however, several also began to surround the injury site. At 2 weeks, astrocytes and microglia then formed a scar (Dusart and Schwab, 1994). This is consistent with contusion injury (Popovich et al., 1997; Zhu et al., 2015b) where an astroglial scar surrounded the lesion, whereas cavitation sites were occupied by microglia and macrophages (Popovich et al., 1997). Interestingly, 1 and/or 4 months after injury, astrocytes expressed several proteins, such as gamma1- and alpha1-laminin, type IV collagen, and FGF2, which participated in the chronic persistence of the glial scar (Liesi and Kauppila, 2002). Likewise, 2 months after the complete section of the thoracic spinal cord, astrocytes produced CSPG in the scar (Li et al., 2018), thus suggesting that, as seen in mice, astrocytes contribute to extracellular matrix dynamics.

From 1 to 7 days following contusion injury, astrocytes, oligodendrocytes, and NG2 glial precursors proliferated in the spared white matter, with a peak on Day 3. About 50% of the astrocytes and oligodendrocytes located in the residual white matter, next to the injury site, however, were lost by 24 h (Zai and Wrathall, 2005). During the chronic phase (6 weeks after lesion), the remaining proliferative cells consist of mature astrocytes or oligodendrocytes (50%) and few expressing NG2 (Zai and Wrathall, 2005). After moderate contusion, the expression level of NG2 increased between 3 and 7 days post injury and remained chronically elevated. In contrast to the spared surrounding tissue, within the lesion site, few, if any, NG2^+^ cells were oligodendrocytes (McTigue et al., 2006). Within areas undergoing Wallerian degeneration, following dorsal funiculotomy, oligodendrocyte density (Olig2) decreased at subacute (10 days) and chronic (30 days) stages, although Olig2^+^ cells were still present (Wang et al., 2009).

Taken together, these results demonstrate that the glial response to SCI exhibits similar dynamics in rats and mice; however, the immune cell response occurs earlier in rats than in mice (Figures 1A,B).

## Nonhuman Primates: A Major Astrocytic Scar Is Not Observed After Spinal Cord Injury

The neuroanatomical organisation of the central nervous system and responses to injury differ between rodents and primates (Courtine et al., 2007); thus, several SCI models in various strains of nonhuman primate have been developed. However, investigation of the glial response following injury is sparse, particularly early after injury (Figure 2 and Table 3).

**TABLE 3:** Studies demonstrating roles of the glial and immune cells after SCI in primates.

**Species, strain, sex, age**	**Interval SCIdeath, methods**	**Injury type, level**	**Astrocyte**	**Microglia/macrophage**	**References**
**Nonhuman primates**					
*Callitrhrix jacchus* (Marmoset), 20F, adults	10wks, IHC	3 contusion severities, C5	GFAP		Iwanami et al., 2005
*Macaca fascicularis*9 M, 5–6yrs	1&4wks, IHC	Lateral HS, T8-9	GFAP	OX42	Shi et al., 2009
*Macacacynomolgus*1M	1hr, IHC	Balloon compression	GFAP	Iba1	Miller et al., 2012
*Macaca fascicularis*4M, 4–6 yrs	7&30dys, IHC	Lateral HS, T8-9	GFAP	Iba1 CD68	Wu et al., 2013
*Callitrhrix jacchus* (Marmoset), 16F, 2yrs	1, 2, 4&6wks, microarrays& RNA-seq. 1, 2&6wks, IHC	Contusion, C5	GFAP	Iba1	Nishimura et al., 2014
*Macaca mulatta* 6M, 3.5–4.2 yrs	6mths, IHC	2 contusion severities, T9	GFAP		Ma et al., 2016
*Chlorocebussabaeus* (african green monkey) 12M, 5–10yrs	12wks, IHC	lateral HS, T9-10	GFAP	Iba1	Slotkin et al., 2017
*Microcebus murinus* 8M, 2 yrs	3mths, IHC	Lateral HS, T12-L1	GFAP	Iba1	Le Corre et al., 2018
*Microcebus murinus* 10M, 2yrs	3mths, IHC	Lateral HS, T12-L1	GFAP	Iba1	Poulen et al., 2021
**Human**					
27 cases, 5F&22M, 8–86 yrs	8 dys-23yrs, IHC	Para- or tetraplegia C, T&L	GFAP		Puckett et al., 1997
13 cases 21–85yrs	2 dys- 30 yrs, IHC	Complete para- or tetraplegia C, T & L	GFAP		Buss et al., 2004
180 cases Ratio 5:1 M:F 8 mths to 92yrs	Instantaneous- 51yrs, IHC&HC	Predominantly C	GFAP	H&E	Norenberg et al., 2004
11 cases, 2F & 9M 18–83yr	30min - 19dys, IHC	Para or tetraplegia.	GFAP	MHCII	Yang et al., 2004
1 case, 56yrs	2yrs IHC	Complete C6 injury	GFAP		Guest et al., 2005
28 cases, 8F&20M, 6–88yrs	Instantaneous - 1yr, IHC	Contusion, compression&lacerationC1-T12.		CD68	Fleming et al., 2006
3 cases, 1F&2M, 49, 59 and 80yrs	15, 20, 60 dys, IHC	Contusion, C		CD68	Chang, 2007
1 case	5dys, IHC		GFAP		Fan et al., 2016
22 cases, 6F&16M 15–80yrs	<1–413 dys, IHC	T & C	IBA1	TMEM119 P2RY12	Zrzavy et al., 2021

*hrs, hours; min, minutes; dys, days; wks, weeks; mths, months; yrs, years; IHC, immunohistochemistry; HC, histochemistry; H&E, hematoxylin eosin; M, male; F, female; C, cervical; T, thoracic; L, lumbar; HS, hemisection.*

One hour after spinal cord compression in *Macaca cynomolgus*, an increased IBA1 immunoreactivity was observed adjacent to the injury site; no modification in astrocytes was seen (Miller et al., 2012). Spatiotemporal investigation of cellular responses following lateral spinal cord hemisection in *Macaca fascicularis* highlighted that, 1 and 4 weeks post-injury, microglia displayed morphological changes and became amoeboid in the epicenter and the spared contralateral white matter (Wu et al., 2013). The number of IBA1 positive cells remained stable at 1 week and decreased 4 weeks after lesion in both locations. However, activated microglia/macrophages (CD68^+^) increased in number at the two time points in the same locations. Concomitantly, at the lesion epicenter, a decreased astrocyte number was reported and astrocytes became hypertrophic contralateral to the lesion. Importantly, a major astrocytic glial scar surrounding the lesion site was never observed (Wu et al., 2013). In the same species and lesion model, 1 and 4 weeks after SCI, an increased number of microglia (OX42^+^) was detected within areas undergoing Wallerian degeneration (Shi et al., 2009). Morphologically, microglia were branched but displayed a large cell body and short processes. None, although, were amoeboid (Shi et al., 2009). No modifications in astrocytic morphology or number were observed.

Longitudinal gene expression analysis following contusion of the cervical spinal cord in *Callithrix jacchus* (marmoset) revealed that the inflammatory response peaked at 1 week post SCI and remained elevated up to 6 weeks following injury (Nishimura et al., 2014). The inflammatory response thus required a longer time to occur than in rodents. Concomitantly, IBA1 positive cells and proliferative microglia were present at the lesion epicenter at 1 week, decreased at 2 weeks, and were absent 6 weeks after injury. The rim of the lesion was delineated by astrocytes only at 6 weeks. In the same species and lesion model but with graded severities, 10 weeks after trauma, GFAP was expressed in a severity-dependent manner at the border of the lesion (Iwanami et al., 2005).

Three months following hemisection of the thoracic spinal cord in *Chlorocebus sabaeus* (African green monkey), astrocytes and microglia/macrophages were present at the rim of the lesion (Slotkin et al., 2017). In *Microcebus murinus*, a small lemur, we have shown that, at 3 months following lateral hemisection of the thoracic spinal cord, the glial reactivity was increased adjacent to the lesion. Additionally, an increase in microglia/macrophage and astrocyte reactivity was present within the grey matter, only rostral to the lesion. Moreover, rostral to the lesion a marked increase in microglia/macrophage reactivity was also observed on the lesion side of the *dorsal funiculus* (Le Corre et al., 2018; Poulen and Perrin, 2018; Poulen et al., 2021).

Finally, in *Macaca mulatta*, 6 months following contusive injury of the thoracic spinal cord, the density of astrocytes was decreased in the lesion penumbra but increased in the spared white matter (Ma et al., 2016).

Overall, the microglial response appears similar as in rodents conversely to the astrocytic response that occurs slower and does not lead to the formation of a major astrocytic scar. In some species, the inflammatory response is also slower than in rats and mice (Figure 1B).

## Human: A Major Astrocytic Scar Is Not Observed After Spinal Cord Injury and Astrocytic Response Is Slower Than in Other Species

Similarly, to animal models of spinal cord injury, microglial/macrophage cells display the earliest cellular response to injury (Figure 2 and Table 3). From 0 to 4 h after injury, a modest number of phagocytic microglia/infiltrating monocyte-derived macrophages were observed at the injury site (Fleming et al., 2006). Activated microglia have also been detected as early as 30 min (Yang et al., 2004) and 1 day (Norenberg et al., 2004; Fleming et al., 2006) after spinal cord injury. Consequently, activated microglia were observed in the surviving area 5 days after SCI (Yang et al., 2004), and numerous amoeboid microglia were present adjacent to areas of necrosis from 5 to 10 days post injury. These persisted for weeks (and up to 1 year) in the proximity of the injury site (Norenberg et al., 2004; Fleming et al., 2006; Chang, 2007). A recent analysis of 22 human SCI cases has highlighted a time-dependent activation of microglia and macrophages associated with a spatial-dependent inflammatory pattern composed of a predominantly pro-inflammatory lesion rim and a lesion core displaying a dual pro- and anti-inflammatory phenotype (Zrzavy et al., 2021). The initial loss of microglia within the core of the lesion at the acute stage (1–3 days post-SCI) was followed in the early subacute stage (4–21 days post-SCI) by a massive increase of IBA1-expressing cells in the core and the rim of the lesion. Within the lesion rim, the majority of these cells were microglia (80% TMEM119^+^) conversely to the core where their proportion dropped to 10% and was associated with an amoeboid shape and a large number of CD68^+^ macrophages. Importantly, IBA1^+^/TMEM119^+^ cells within the lesion rim mostly resulted from local microglial proliferation (Zrzavy et al., 2021). Later (21–90 days post-injury), the number of macrophages in the lesion core decreased but remained elevated and displayed a dispersed pattern in the lesion rim. At chronic stages (90 days to 1.5 years post-lesion), cystic cavitations appeared and were surrounded by a rim of activated astrocytes and macrophages. Overall, microglia are, thus, the predominant cells in the proximity of the injury during lesion maturation, and recruited monocytes/macrophages are dominant within the lesion core.

Only a few studies have investigated the temporal astrocytic response following spinal cord injury in man. The presence of activated astrocytes has been described to appear either early after the injury (from 4 days) (Buss et al., 2004; Norenberg et al., 2004) at 21–90 days post-injury in the lesion rim (Zrzavy et al., 2021) or as long as 4 months after lesion (Puckett et al., 1997). In one spinal cord sample, 5 days after SCI, necroptotic markers were found in GFAP^+^ cells located in the lesion site, suggesting that reactive astrocytes may undergo necroptosis (Fan et al., 2016). Clusters of activated astrocytes were also observed one or two segments away from the lesion site in both white and grey matters from 4 to 12 days after SCI. Thereafter, activated astrocytes were evenly distributed over the whole section close to the lesion site from 24 days to 4 months after injury (Buss et al., 2004). Several days after injury, hypertrophic astrocytes appeared at the edge of the lesion and peaked at 2–3 weeks (Norenberg et al., 2004). Moreover, activated astrocytes surrounded cystic cavities from 90 days to 1.5 years post injury (Zrzavy et al., 2021). In another study, astrocytes displayed a slight increase in GFAP reactivity, in processes in contact with the phagocytes, 4 to 12 months after injury, followed by a hypointense GFAP signal, persisting up to 23 years after injury (Puckett et al., 1997). At longer post-injury time (1–30 years), dense GFAP-positive staining was present in the white matter that had undergone Wallerian degeneration (Buss et al., 2004). Two years following complete spinal cord injury, a dense GFAP reaction was observed in the peri-injury region (Guest et al., 2005). These differences may result from the heterogeneity of the lesions observed in man.

Overall, the astrocytic response in man seems to occur slower than in animal models, including nonhuman primates, and the astroglial processes that create an impenetrable barrier were almost never seen (Norenberg et al., 2004) (Figures 1B, 2).

## Species With High Regenerative Capacities: A Glial Bridge More Than a Glial Scar

**FIGURE 3:**
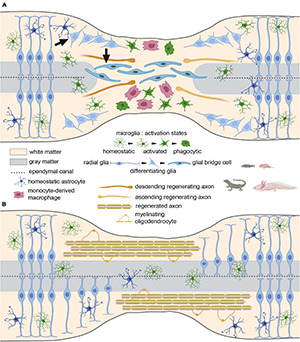
The glial bridge after spinal cord injury in species with high regenerative capacities and perinatal mammals. **(A)** Tissue clearance, glial bridge, and axon sprouting at the acute/subacute stage. Arrows represent the involvement of radial glia in the glial bridge formation. **(B)** Remyelination and return to homeostasis at the chronic stage.

Interestingly, vertebrates, such as fishes, urodele amphibians, and some reptiles, that possess a remarkable capacity to regenerate injured spinal cord tissue and to recover associated functions also display rather limited (if any) glial scarring (Figure 3 and Table 4). Radial glial cells are the main (and often the only) representant of astrocytes in lower vertebrates (for review, see Verkhratsky et al., 2019). Here, we kept the names “radial glia,” “GFAP-expressing cells” or even “astrocytes” as they appeared in the original publications.

**TABLE 4:** Studies demonstrating roles of the glial and immune cells after SCI in species with high regenerative capacities.

**Species, injury, interval SCI-death, methods**	**Astrocyte**	**Radial cells**	**Microglia/macrophage**	**Infiltrating cells & other glia**	**References**
**Regenerate embryonic/larvae**					
Rats: Adults *vs* E19, FT.T8-10. 3, 7, 21&35dys.IHC, ISH	GFAP	OX42			Fujimoto et al., 2006
Zebra larvae 5dpf. FT. ISH, IH	GFAP	Tg Dbx1a,		Tg olig2	Briona and Dorsky, 2014
Rats: Adult *vs* E18. FT. T9-10. *Gekkos japonicus*, FT. L10-11. 1& 4wks. IHC	GFAP				Gu et al., 2015
Zebra larvae. 2dpf Mechanical lesion. IHC			L-Plastin 4C4	Tg olig2	Ohnmacht et al., 2016
Zebra larvae. FT. ISH, IHC.			Tg: *mpeg1*/4C4^+^ *mpeg1*/4C4^–^	TgMpx	Tsarouchas et al., 2018
Zebra larvae. FT. BrdU.			Tg mpeg	Tg: Mpx olig2	Anguita-Salinas et al., 2019
Neonate rats Zebra larvae. 3dpf. Dexamethasone FT.6, 24, 48, 72&120 hrs				Tg *cloche* Nr3c1 GFAP	Nelson et al., 2019
Neonate mice P2. Crush. ISH, IHC, RNAseq	GFAP		CX3CR1 Csf1r^*flox*,^ CD68, P2RY12		Li et al., 2020
*Xenopuslaevis* Regenerative and non-regenerative stages. FT. EM, IHC	Vimentin BLBP GS				Edwards-Faret et al., 2021
Zebra larvae. 3dpf. Stab injury. 12hrs. FACS, RNAseq. IHC.	Tg GFAP			SOX2 NG2	Zeng et al., 2021
**Regenerate adults**					
*Amybstoma mexicanum* (axolotl) Juvenile and adult. FT. 1, 2, 3, 4, 5&6wks. EM. IHC.	GFAP				O’Hara et al., 1992
Zebrafish, FT. IHC, EM			4C4		Becker and Becker, 2001
Newts (Salamander), FT. 1&3 dys; 1, 2, 3, 6&9 wks. IHC, HC, EM.	GFAP				Zukor et al., 2011
Zebrafish, FT. BrdU, IHC	GFAP, vimentin				Goldshmit et al., 2012
Zebrafish, FT.IHC, ISH, tissue clearing, EdU	GFAP			Tg(olig2:eGFP	Tsata et al., 2020

*FACS, flow cytometry; hrs, hours; dys, days; wks, weeks; IHC, immunohistochemistry; T, thoracic; L, lumbar; FT, full transection; EM, electronic microscopy; ISH, *in situ* hybridisation; dpf, day post fertilisation; Tg, transgenic; E, embryonic.*

No reactive, fibrous astrocytes have been described at the injury site, after spinal cord lesion, in either juvenile or adult *Amybstoma mexicanum* (axolotl). Astrocytes, initially present in the white matter, first disappeared from the lesion site and reappeared 1 month after injury concomitantly with regenerating axons (O’Hara et al., 1992). In the same study, *in vitro* experiments suggested that the formation of a scaffold resulting from mesenchymal epithelial transition permits axon regeneration (O’Hara et al., 1992). Similarly, in the adult salamander, following the complete spinal cord section, axons regrew and crossed the lesion site (Zukor et al., 2011). No scar formation was observed; however, astrocytes were present but not hypertrophic. Astrocytic cells did not migrate into the injury site, but GFAP^+^ processes crossed the lesion site, and axons appeared to regrow on this glial support. Additionally, a non-detrimental inflammatory response was reported (Zukor et al., 2011). Likewise, following SCI in adult zebrafishes (Goldshmit et al., 2012) and larvae (Briona and Dorsky, 2014), GFAP-expressing cells became elongated and formed a “glial bridge” that joins the sides of the damaged spinal cord in the absence of glial scar formation. No reactive astrocytes were observed. In adults, within 3–5 days post injury, GFAP^+^ glial cells proliferated in and around the central canal. Concomitantly, a few proliferative macrophages were also reported outside of the central canal (Goldshmit et al., 2012). Five days after injury, proliferative cells at the edge of the lesion expressed a low level of GFAP, and, from 7 to 10 days after SCI, GFAP^+^ cells migrated into the site of the lesion and acquired a bipolar morphology. Then, from 2 to 3 weeks post SCI, a “glial bridge” formed of GFAP-expressing bipolar cells appeared in the lesion site. From 4 weeks post lesion, this permissive bridge supported axogenesis. Interestingly, by 3 (and up to 5) days post injury, oligodendrocyte precursors and motor neuron progenitors (olig2^+^) bridged the injury site in zebrafish larvae (Anguita-Salinas et al., 2019). The mechanisms of bridge formation appeared to be Fgf- (Goldshmit et al., 2012) but also ctfg (connective tissue growth factor) dependent (reviewed in Cigliola et al., 2020). In zebrafish, bridge formation depends on the proliferation of ependymal glia. Remarkably, glucocorticoids directly inhibited the formation of *trans*-lesion glial bridges and prevented axon regrowth and functional recovery through activation of Nr3c1 signalling (Nelson et al., 2019). There is still debate as to whether the glial bridge is prerequisite to axonal regrowth or whether it forms concomitantly with regenerating axons (reviewed in Cigliola et al., 2020). Additionally, in the larval zebrafish, Dbx1a-expressing cells that persist as radial glia and represent a pool of neurogenic progenitors can be activated in response to injury and differentiate into neurons (Briona and Dorsky, 2014). In early developmental stages, radial glial cells displaying a bipolar shape are abundantly present in both mammals and salamanders. Following SCI in salamander, radial glia cells ligate both rostral- and caudal-sectioned ends of the spinal cord before proliferating and differentiating into other glial cells (including astrocytes and oligodendrocytes) and into neurons (reviewed in Tazaki et al., 2017). Along this line, in zebrafish embryos, stress-responsive regenerating cells that are induced by SCI and that play an essential role in axonal regeneration have been identified and further characterised as mostly composed of radial glia (Zeng et al., 2021). In contrast, upon SCI, radial glial cells of adult mammalians generate astrocytes. Instead of stretching to build an ependymal bridge, these astrocytes participate in the formation of a glial scar and prevent axonal regeneration. Further experiments to investigate the role of radial glia in neonatal mammals after SCI would certainly provide interesting findings to develop therapeutic strategies to favour axonal regeneration.

In both adult and larval zebrafish, the recruitment of immune cells has been observed after SCI. In adults, reactive microglia were observed at 2–3 days and at 14 days after spinal cord injury (Becker and Becker, 2001). In zebrafish larvae, recruitment of immune cells was observed as early as 2 h following the complete spinal cord section with a peak of neutrophils accumulation at the injury site (Tsarouchas et al., 2018). A slightly different time window of activation has also been reported after the complete spinal cord section, with a strong neutrophil recruitment until 12 h post injury at the lesion site, followed by its disappearance 24 h post injury (Anguita-Salinas et al., 2019). Macrophages and microglia were reported to be increased at 48 h post injury (Ohnmacht et al., 2016; Tsarouchas et al., 2018; Anguita-Salinas et al., 2019) and were detected next to the transection site at 7 and 42 days post lesion (Tsata et al., 2020). A brief, pro-inflammatory macrophage response, followed by an anti-inflammatory state, was observed that may underlie rapid myelin debris clearance (reviewed in Ghosh and Hui, 2018) and has led to the hypothesis of a similarity between peripheral nervous system injury in mammals and CNS injury in zebrafish (Ghosh and Hui, 2018). Moreover, following the complete section of the adult zebrafish spinal cord, oligodendrocyte precursor cells survived, proliferated, and replaced lost oligodendrocytes that reestablished myelination (Tsata et al., 2020).

Comparison between regenerative (pre-metamorphosis stages) and non-regenerative (during metamorphosis) responses in *Xenopus laevis* highlighted that, in the same species, no glial scar was observed in regenerative stages conversely to the non-regenerative stage where a transient glial scar-like structure was formed (Edwards-Faret et al., 2021). Similarly, spinal crush injury in neonatal mice up to postnatal Day 2 led to scar-free healing, allowing axonal regrowth through the lesion (Li et al., 2020). When SCI occurred at 2 days post-natal (regenerative response), amoeboid activated microglia first accumulated in the stumps 2–3 dpi and quickly returned to a ramified “resting” morphology by 2 weeks post-injury, when spinal cord regeneration was complete. When SCI occurred after 7 days post-natal (the non-regenerative stage), microglia remained highly activated for at least 2 weeks. Moreover, RNA sequencing at 2 days post-natal highlighted that this transient microglial activation permitted the formation of a temporary fibronectin bridge that ligated the two ends of the spinal cord and allowed axon regeneration (Li et al., 2020). Likewise, an intra-uterine complete section of the spinal cord at embryonic Day 19 in rats led to an absence of glial scar formation conversely to the same injury in adults (Fujimoto et al., 2006). Time course analysis showed an increase in the number of astrocytes and microglia/macrophages (OX42^+^) in adults from 3 to 35 days after injury. Conversely, fetal injury led to a transient and rather limited increase in the number of astrocytes and microglia/macrophages at 3 and 3–7 days after injury, respectively. Additionally, leucocyte and macrophage infiltration were reported 3 and 7 days after SCI only in adults. In rodents, fetal and postnatal Day 2 injury thus led to a transient and limited activation of glial cells in the surrounding of the lesion contrariwise to SCI at the adult stage.

Comparative studies have been carried out in species, displaying high and low regenerative capabilities. One study characterised GFAP expression, following the complete spinal cord section in the adult gecko (*Gekko japonicum*), a reptile that displays a remarkable capacity for tail restoration, and adult rats. Concomitantly, astrocytic response was compared, following an *in vitro* scratch assay in adult geckos and rats and embryonic rats (Gu et al., 2015). In adult rats, GFAP expression was continuously increased from 1 to 4 weeks after SCI, while geckos displayed a transient expression peak at 1 week, followed by a decrease at 4 weeks. Moreover, astrocytes subjected to *in vitro* scratch wound displayed a higher GFAP expression and higher proliferative ability in adult rats than in embryonic rats and adult geckos. Lastly, it has been demonstrated, in zebrafish and rat, that the opposing regulation of the ependymal glial glucocorticoid receptor (Nr3c1), after complete spinal cord injury, participated in the differential responses between species (Nelson et al., 2019).

Taken together, these studies demonstrate that glial cells are present after spinal cord injury in non-mammal species and mammalian developmental stages that display spinal cord regeneration but respond differently as compared to the adult mammalian nervous system and seem to favour axon regeneration instead of hindering regrowth (Figure 3).

## Concluding Comments and Future Directions

Responses of glial and immune cells following spinal cord injury display similarities and differences across species that are strongly correlated with functional recovery. The overall dynamics of the glial response to SCI in adult rodents and primates, which present extremely limited tissue repair and functional recovery, is comparable across species. Indeed, at acute and subacute stages, an early activation of microglia/macrophages precedes immune-cell infiltration and astrocyte activation (Figures 1A,B, 2A). Both microglia/macrophages and astrocytes proliferate and migrate toward the lesion site. At later stages, astrocytes form an astroglial barrier that surrounds the lesion core. Microglia and NG2 cells also constitute the stabilised scar with tight interlacing between all cell populations (soma and processes) (Figure 2B). The core of the lesion is composed of a fibrotic scar with monocyte-derived macrophages, infiltrating immune cells and a few activated microglia (Figure 2B).

Strikingly, temporal dynamics and levels of activation differ across species. In rodents, rats exhibit an earlier and monophasic infiltration of immune cells conversely to mice that display a delayed biphasic neutrophil and T cell infiltration (Figure 1A). Interestingly, in man, the peak number of neutrophils bears more similarity to mice than rats (Mawhinney et al., 2012). Another major difference is that cystic cavities are observed only (or at least predominantly) in rats and primates. Moreover, in primates, the astrocytic response is delayed and displays a lower level of activation as compared to rodents (Figure 1B).

The dynamics of the glial response to SCI is different in non-mammal species/mammalian developmental stages that exhibit high regenerative capacities and functional recovery. In particular, astroglial activation differs drastically, since astrocytes migrate toward the lesion site but form a bridge (Figure 3), and not a scar (Figure 2), which permits axonal regrowth through the lesion site. Interestingly, similar mechanisms are observed in embryonic and fetal mammals. The inflammatory response to SCI seems slightly different and leads to a faster myelin clearance that may resemble peripheral nervous system injury in adult mammals.

Recent findings have highlighted a sexual dimorphism in glial and immune cell responses present in pain signalling (for review, see Midavaine et al., 2021); thus, future investigations of the sex-dependent glial response and its crosstalk with immune cells, following SCI, are of great interest. The analysis of cellular dynamics following SCI in different contexts (species, age, sex, etc.) will help in the design of efficient therapeutic strategies used concomitantly, or sequentially, to improve recovery after CNS lesion.

## Author Contributions

J-CP participated in the design of the review, analysed the data, and prepared the figures. YG contributed to the design of the review and the analysis of the data. FP conceptualised the design of the review, participated in the analysis and data interpretation, wrote the manuscript, and approved the final review. All authors contributed to the article and approved the submitted version.

## Conflict of Interest

The authors declare that the research was conducted in the absence of any commercial or financial relationships that could be construed as a potential conflict of interest.

## Publisher’s Note

All claims expressed in this article are solely those of the authors and do not necessarily represent those of their affiliated organizations, or those of the publisher, the editors and the reviewers. Any product that may be evaluated in this article, or claim that may be made by its manufacturer, is not guaranteed or endorsed by the publisher.
